# Extended-spectrum β-lactamase, plasmid-mediated AmpC β-lactamase, fluoroquinolone resistance, and decreased susceptibility to carbapenems in *Enterobacteriaceae*: fecal carriage rates and associated risk factors in the community of Northern Cyprus

**DOI:** 10.1186/s13756-019-0548-9

**Published:** 2019-06-10

**Authors:** Emrah Ruh, Jonathan Zakka, Kujtesa Hoti, Arezou Fekrat, Emrah Guler, Umut Gazi, Zafer Erdogmus, Kaya Suer

**Affiliations:** 1 Department of Medical Microbiology and Clinical Microbiology, Faculty of Medicine, Near East University, Nicosia, Northern Cyprus; 2 Department of Infectious Diseases, Dr. Burhan Nalbantoglu State Hospital, Nicosia, Northern Cyprus; 3 Department of Clinical Microbiology and Infectious Diseases, Faculty of Medicine, Near East University, Nicosia, Northern Cyprus

**Keywords:** *Enterobacteriaceae*, Fecal carriage, Antibiotic resistance, Extended-spectrum beta lactamase, Plasmid-mediated AmpC beta-lactamase, Ciprofloxacin resistance, Carbapenem resistance, Northern Cyprus

## Abstract

**Background:**

Antibiotic-resistant *Enterobacteriaceae* in the gastrointestinal flora can lead to infections with limited therapeutic options. Also, the resistant bacteria can be transferred from colonized persons to others. The present study was conducted to search the fecal carriage rates of (i) *Enterobacteriaceae* that produce extended-spectrum β-lactamase (ESBL-E) and/or (ii) plasmid-mediated AmpC β-lactamase (pAmpC-E), (iii) ciprofloxacin-resistant *Enterobacteriaceae* (CIP-RE), and (iv) carbapenem-intermediate or -resistant *Enterobacteriaceae* (CIRE) in Northern Cyprus.

**Methods:**

A total of 500 community-dwellers were recruited from consecutive admissions to the clinical laboratories of four hospitals. One rectal swab or stool sample was collected from each participant. A questionnaire was applied to evaluate possible risk factors associated with intestinal colonization of resistant bacteria. The samples were cultured on antibiotic containing media to screen for resistant bacteria colonization. The bacterial colonies that grew on the plates were subjected to further phenotypic tests to confirm the resistance.

**Results:**

Of 500 volunteers, ESBL-E, pAmpC-E, CIP-RE and CIRE carriage were detected in 107 (21.4%), 15 (3.0%), 51 (10.2%) and six (1.2%) participants, respectively. *Escherichia coli* was the most commonly recovered species among *Enterobacteriaceae* isolates. A significant proportion of ESBL-producing *E. coli* isolates (*n* = 22/107; 20.6%) was found to be co-resistant to CIP (*p* = 0.000, OR 3.21, 95% CI 1.76–5.87). In this study, higher socioeconomic status (CIP-RE: *p* = 0.024, OR 1.96, 95% CI 1.09–3.53), presence of gastrointestinal symptoms (CIRE: *p* = 0.033; OR 6.79, 95% CI 1.34–34.39), antibiotic use (ESBL-E: *p* = 0.031; OR 1.67, 95% CI 1.04–2.67; and CIRE: *p* = 0.033; OR 6.40, 95% CI 1.16–35.39), and travelling abroad (pAmpC-E: *p* = 0.010; OR 4.12, 95% CI 1.45–11.66) were indentified as risk factors.

**Conclusion:**

The study indicates that resistant *Enterobacteriaceae* isolates are carried by humans in the community. To prevent further spread of resistance, rational use of antibiotics should be encouraged, and antibiotic resistance should be carefully monitored in Northern Cyprus.

## Background

*Enterobacteriaceae* are gram-negative bacteria that are members of normal intestinal flora. Also, these bacteria are the most common cause of infections in hospital and community settings [1]. More importantly, increasing rates of antibiotic resistance in *Enterobacteriaceae* are reported globally [2].

Extended-spectrum β-lactamases (ESBLs) are the primary reason of acquired drug resistance seen in gram-negative bacteria [3]. ESBLs have been a global concern, and the resistance rates have increased [4]. ESBL enzymes can hydrolyze many β-lactam antibiotics except for cephamycins and carbapenems [5]. Apart from ESBLs, production of AmpC β-lactamases is the other mechanism that confers resistance to broad-spectrum cephalosporins [6]. Importantly, these enzymes also have the ability to hydrolyze cephamycins [7].

Carbapenems are the antibiotics of last resort that are used for the treatment of multidrug-resistant *Enterobacteriaceae* (MDR-E) infections [8]. However, carbapenem-intermediate or -resistant *Enterobacteriaceae* (CIRE) have spread across the globe [9]. Carbapenem resistance in *Enterobacteriaceae* occur mainly due to the production of carbapenemase enzymes in bacteria [10]. Carbapenemase production, except for OXA-48, is associated with increased resistance against most of the β-lactams; while activity of the enzymes on carbapenems is variable [11].

Fluoroquinolones (FQs) are commonly used for the therapy of various infections [12]. These antibiotics are also one of the treatment options for ESBL-positive infections [13]. However, FQ resistance is reported at high rates among ESBL-producing *Enterobacteriaceae* (ESBL-E) species [14].

The intestinal tract is the primary source of *Enterobacteriaceae*, and moreover, transfer of antibiotic resistance genes occur here, and resistant bacteria proliferate as a result of antibiotic therapy [15]. Therefore, resistant enteric bacteria in the gut flora can lead to infections with limited therapeutic options. Furthermore, resistance genes of the intestinal microbiota can spread from one person to another, and to the environment [16].

Fecal carriage of ESBL-E, plasmid-mediated AmpC β-lactamase-producing *Enterobacteriaceae* (pAmpC-E), FQ-resistant *Escherichia coli*, or carbapenem-resistant *Enterobacteriaceae* (CRE) has been demonstrated by a number of research studies [4, 6, 17–20]. Moreover, several factors including previous exposure to antibiotics have been associated with an increased risk of fecal carriage of resistant *Enterobacteriaceae* [4, 20–22].

In the literature, there is no published data on the intestinal colonization of resistant enteric bacteria in Northern Cyprus. Nevertheless, several studies indicated high rates of antibiotic resistance in *Enterobacteriaceae*. Indeed, the percentages of ESBL-producing *E. coli* were detected to be 53 and 44% in the urine samples of hospitalized and outpatients, respectively. Importantly, FQ resistance among ESBL-producing *E. coli* isolates was found to be 78 and 79% in the samples of inpatients and outpatients, respectively. ESBL-positive *E. coli* isolates remained susceptible to imipenem (IMP) and meropenem (MEM), however ertapenem (ERT) resistance was noted to be 6% in hospitalized patients, and 11% in outpatients [23]. Another hospital-based study in Northern Cyprus documented the rate of ESBL to be 16.7% in *Klebsiella pneumoniae* isolates. Moreover, FQ resistance was reported between 16.8 and 20.1%, whereas IMP, MEM and ERT resistance rates were found to be 0.0, 1.0, and 4.6%, respectively, among *K. pneumoniae* isolates [24].

In the light of above facts, the present study aimed to determine the fecal carriage rates of ESBL-E, pAmpC-E, ciprofloxacin-resistant *Enterobacteriaceae* (CIP-RE), and CIRE among community-dwelling healthy individuals in Northern Cyprus. Additionally, possible risk factors associated with intestinal colonization of ESBL-E, pAmpC-E, CIP-RE and CIRE were evaluated. To our knowledge, this is the first study that presents the fecal carriage rates of resistant *Enterobacteriaceae* and associated risk factors in Northern Cyprus.

## Methods

### Study population

Between September and December 2017, volunteers for this study were recruited from consecutive admissions to the routine clinical laboratories of Near East University Hospital (Nicosia), Nicosia Dr. Burhan Nalbantoglu State Hospital, Kyrenia Dr. Akcicek Hospital and Famagusta State Hospital. Subjects who were referred to the laboratories for stool examination as a part of routine health check-up were informed about the study. The inclusion criteria were being older than 18 years of age, living in Northern Cyprus for at least one year, and not being hospitalized. Eventually, a total of 500 community-dwelling individuals who met these criteria were enrolled in this study.

### Ethical approval

The ethical approval for the study was obtained from the Near East University Research Assessment Committee (Project no: YDU/2016/37–296). Written informed consent was collected from each participant.

### Sample and data collection

From each participant, one rectal swab or stool sample was collected, therefore a total of 500 specimens were included in the study. During the sample collection, a questionnaire was applied to each participant in order to assess the possible risk factors for fecal carriage of resistant *Enterobacteriaceae*. In the questionnaire, demographic and socioeconomic data (age, gender, level of education, and economic status) were recorded. Next, the participants were asked whether they had at least one gastrointestinal symptom (GIS) (diarrhea, constipation, abdominal cramps, nausea or bloating) during the sample collection. Participants also provided information on history of antibiotic use and travelling abroad within six months from sample collection. In the data, there were missing information regarding the antibiotic names, therefore, these were excluded from the analysis to rule out inaccurate evaluation.

### Phenotypic screening for ESBL-E, pAmpC-E, CIP-RE, and CIRE isolates

Fecal swabs or stool samples (200 mg) were initially suspended in 2 ml of sterile 0.9% saline. Next, an aliquot of each sample were plated onto four sets of MacConkey media (Merck, Germany): (i) control plate without antibiotics, (ii) biplate agar containing cefotaxime (CTX) (Sigma, USA) and ceftazidime (CAZ) (Sigma, USA) at 1 mg/L concentration to screen for ESBL- and/or pAmpC-producing isolates [17], (iii) medium containing ciprofloxacin (CIP) (Sigma, USA) at 1 mg/L to screen for FQ-resistant isolates [18], and (iv) medium supplemented with 1 mg/L ertapenem (ERT) (Sigma, USA) to screen for carbapenem-resistant strains [25, 26]. All of the plates were incubated overnight at 37 °C.

### Phenotypic confirmatory tests and identification of ESBL-E, pAmpC-E, CIP-RE, and CIRE isolates

Bacterial isolates that grew on CTX and/or CAZ containing media were further tested for the presence of ESBL- and/or pAmpC-production. Confirmation of ESBL-producing isolates was done by using the combined disc test, according to the Clinical and Laboratory Standards Institute (CLSI) guidelines [27]. CTX (30 μg) and CAZ (30 μg) discs with or without clavulonic acid (CLA) (10 μg) were used. An increase of ≥5 mm in the zone diameter of CTX/CLA or CAZ/CLA compared to the antibiotics alone was considered positive for ESBL production [27]. For the initial screening of pAmpC activity, all the isolates that grew in CTX and/or CAZ containing media were tested using cefoxitin (FOX) (30 μg) discs [6]. The isolates with reduced susceptibility (zone diameter of < 18 mm) to FOX [27] were further analyzed by combined disk (CD) method. CTX (30 μg) and CAZ (30 μg) discs were used with or without cloxacillin (750 μg), an inhibitor of pAmpC. An increase of ≥5 mm of the zone diameter in the presence of the inhibitor was interpreted as a positive pAmpC test [17].

In order to confirm FQ resistance, bacterial strains recovered from CIP containing plates were subjected to disc diffusion test using CIP (5 μg), ofloxacin (OFX) (5 μg), norfloxacin (NOR) (10 μg), levofloxacin (LVX) (5 μg) and gemifloxacin (GEM) (5 μg) discs. Inhibition zones of ≤15 mm for CIP and GEM; ≤12 mm for OFX and NOR; and ≤ 13 mm for LVX discs were interpreted as resistant [27].

The isolates that grew in ERT plates were evaluated by disc diffusion test using ERT (10 μg), MEM (10 μg) and IMP (10 μg) discs. Inhibition zones of ≤18 mm for ERT disc; and ≤ 19 mm for IMP and MEM discs indicated resistance [27]. The isolates with confirmed resistance to carbapenems were further tested for carbapenemase activity. Presence of metallo-β-lactamase (MBL) was assessed by combined disc method using ERT (10 μg) disc alone and with 0.1 M EDTA (10 μl). An increase of ≥5 mm in the zone diameter of ERT/EDTA compared to ERT alone was recorded as MBL-positive [28]. *Klebsiella pneumoniae* carbapenemase (KPC) detection was done by double disc synergy test (DDST) using ERT (10 μg) and boronic acid (300 μg) discs. An increase in the zone diameter of ERT towards boronic acid disc was interpreted as a positive result for KPC [29]. All antibiotic discs used in this study were purchased from Bioanalyse (Turkey).

**Fig. 1:**
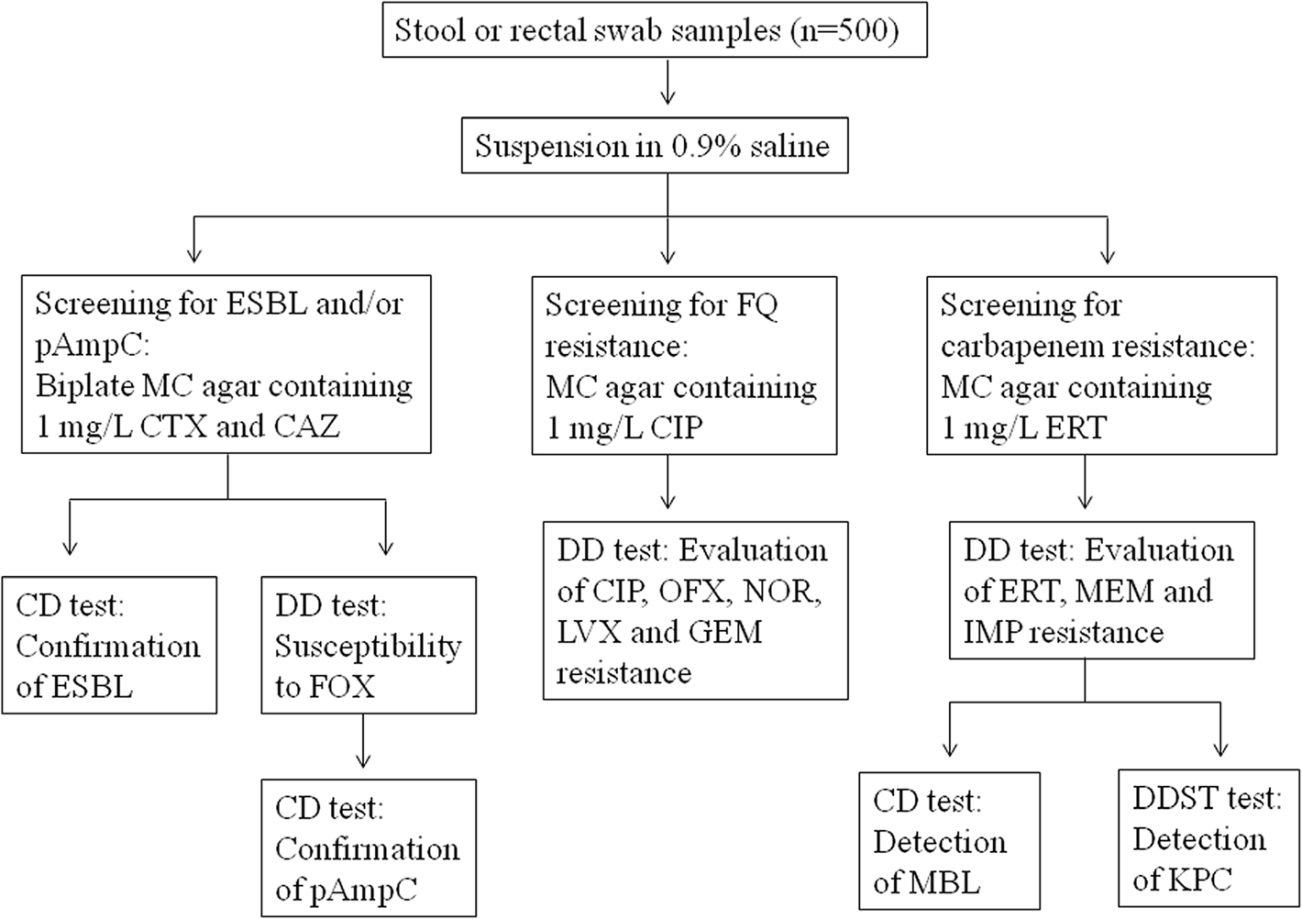
Flow chart of the study protocol. Abbreviations: ESBL, extended-spectrum β-lactamase; pAmpC, plasmid-mediated AmpC β-lactamase; FQ, fluoroquinolone; MC agar, MacConkey agar; CTX, cefotaxime; CAZ, ceftazidime; CIP, ciprofloxacin; ERT, ertapenem; CD test, combined disc test; DD test, disk diffusion test; FOX, cefoxitin; OFX, ofloxacin; NOR, norfloxacin; LVX, levofloxacin, GEM, gemifloxacin, MEM, meropenem; IMP, imipenem; MBL, metallo-β-lactamase; DDST test, double disc synergy test; KPC, *Klebsiella pneumoniae* carbapenemase

Identification of ESBL-E, pAmpC-Ε, CIP-RE, and CIRE isolates was done by using the BD Phoenix 100 system (software version 6.01A). The flow chart of the study protocol is given in the Fig. 1.

### Statistical analysis

Descriptive statistics of the variables in the questionnaire were calculated. For categorical variables, frequency and percentage information were given while for the continuous variables arithmetic mean, standard deviation, median, minimum and maximum were calculated. Depending on the sample sizes, either Pearson Chi-square or Fisher’s exact test was applied for evaluating the associations between categorical variables. For risk assessments of independent variables, odds ratios (OR) with 95% confidence intervals (95% CI) were calculated. All statistical calculations were performed with IBM SPSS statistics package for Macintosh (Demo version 22.0; Armonk, NY: IBM Corp.). Level of significance was accepted as 0.05.

## Results

### General characteristics of the study population

A total of 500 volunteers were recruited from Near East University Hospital (*n* = 49; 9.8%), Nicosia Dr. Burhan Nalbantoglu State Hospital (*n* = 39; 7.8%), Kyrenia Dr. Akcicek Hospital (*n* = 383; 76.6%) and Famagusta State Hospital (*n* = 29; 5.8%). Fecal swab (*n* = 448; 89.6%) or stool (*n* = 52; 10.4%) samples were collected from the participants and included for microbiological analysis. Out of 500 individuals, 320 (64.0%) were male, and 180 (36.0%) were female. The mean and median age of the study population were 32.92 ± 9.97 and 31.00 (19.00–78.00), respectively. Distribution of the participants according to the age groups was 238 (48.2%) for age 19–30, and 256 (51.8%) for age 31 and above. One hundred and eighteen (23.6%) of the individuals had a university or higher degree diploma. Number of the participants with middle or high income was 194 (39.4%).

Out of 500 study participants, 67 (13.4%) presented with one or more GIS at the time of sample collection. One hundred and twenty-four (24.8%) individuals declared that they used antibiotic within six months from the study. Ninety-two (18.4%) persons travelled out of Northern Cyprus within the last six months. Of 85 individuals who provided information on the country visited, 11 (12.9%) travelled to countries in Asia (*n* = 8) or Africa (*n* = 3), while 74 (87.1%) visited other regions (Turkey, *n* = 65; Europe, *n* = 8; Australia, *n* = 1) in the six months before the study.

### Gastrointestinal colonization with ESBL-E, pAmpC-E, CIP-RE, and CIRE

In the present study, 107 (21.4%) of 500 individuals were found to be colonized with ESBL-E. *E. coli* accounted for the most ESBL cases (*n* = 101/107; 94.4%), while *K. pneumoniae* (*n* = 3/107; 2.8%), *Enterobacter cloacae* (*n* = 1/107; 0.9%), *Enterobacter aerogenes* (*n* = 1/107; 0.9%), and *Providencia rettgeri* (*n* = 1/107; 0.9%) were detected at low rates. Fifteen (3.0%) of 500 participants were colonized with pAmpC-E, and the isolates were identified as *E. coli* (*n* = 13/15; 86.7%) and *K. pneumoniae* (*n* = 2/15; 13.3%). Fecal carriage rate of CIP-RE was detected to be 10.2% (*n* = 51/500), and all of the 51 (100%) isolates were defined as *E. coli*. Among CIP-resistant *E. coli* strains, number of the isolates which were resistant to OFX, NOR, LVX and GEM was 35 (68.6%), 36 (70.6%), 31 (60.8%) and 22 (43.1%), respectively. In the study, six (1.2%) out of 500 participants were colonized with CIRE. These isolates were identified as *E. coli* (*n* = 2/6; 33.3%), *K. pneumoniae* (*n* = 2/6; 33.3%), *K. oxytoca* (*n *= 1/6; 16.7%) and *E. aerogenes* (*n* = 1/6; 16.7%). One of the *E. coli* isolates was ERT-resistant, MEM-susceptible and IMP-intermediate. This isolate was found to harbour KPC type carbapenemase by DDST. *K. oxytoca* isolate showed resistance against ERT, MEM and IMP. In this isolate, MBL phenotype was detected by CD test. Other four isolates were intermediately resistant to ERT, and susceptible to both MEM and IMP. Distribution of bacterial species among ESBL-E, pAmpC-E, CIP-RE and CIRE isolates is shown in Table 1.

**Table 1:** Distribution of bacterial species among ESBL-E, pAmpC-E, CIP-RE and CIRE isolates, Northern Cyprus, 2017

Bacterial species	ESBL-E	pAmpC-E	CIP-RE	CIRE
	n/N (%)^a^	n/N (%)^a^	n/N (%)^a^	n/N (%)^a^
*Escherichia coli*	101/107 (94.4)	13/15 (86.7)	51/51 (100.0)	2/6 (33.3)
*Klebsiella pneumoniae*	3/107 (2.8)	2/15 (13.3)	–	2/6 (33.3)
*Klebsiella oxytoca*	–	–	–	1/6 (16.7)
*Enterobacter cloacae*	1/107 (0.9)	–	–	–
*Enterobacter aerogenes*	1/107 (0.9)	–	–	1/6 (16.7)
*Providencia rettgeri*	1/107 (0.9)	–	–	–
Total n/N (%)^b^	107/500 (21.4)	15/500 (3.0)	51/500 (10.2)	6/500 (1.2)

Abbreviations: *ESBL-E* Extended-spectrum β-lactamase-producing *Enterobacteriaceae*; *pAmpC-E* plasmid-mediated AmpC β-lactamase-producing *Enterobacteriaceae*, *CIP-RE* Ciprofloxacin-resistant *Enterobacteriaceae*, *CIRE* Carbapenem-intermediate or -resistant *Enterobacteriaceae*

^a^Indicates the percentage of bacterial species among ESBL-E (*N* = 107), pAmpC-E (*N* = 15), CIP-RE (*N* = 51), or CIRE (*N* = 6) isolates

^b^Indicates the percentage of ESBL-E, pAmpC-E, CIP-RE and CIRE isolates according to 500 samples

Of 500 samples tested, 22 (4.4%) ESBL-producing *E. coli* isolates were found to be co-resistant to CIP. Among these, two (0.4%) *E. coli* isolates were additionally pAmpC producers. Association of CIP resistance and ESBL-production (*n* = 22/107; 20.6%) in *E. coli* strains was found to be statistically significant (*p* = 0.000, OR 3.21, 95% CI 1.76–5.87). In the study, another pAmpC-positive *E. coli* isolate (*n* = 1; 0.2%) showed resistance to CIP. Also, one (0.2%) pAmpC-positive *E. coli* isolate was intermediately resistant to CIP. One (0.2%) *E. coli* and one (0.2%) *K. pneumoniae* isolate were ESBL-positive and ERT-intermediate (MER- and IMP-susceptible). One (0.2%) *K. pneumoniae* isolate was both ESBL- and pAmpC-positive, CIP-intermediate, and also ERT-intermediate (MER- and IMP-susceptible). Furthermore, a sample grew two isolates: one (0.2%) CIP-resistant *E. coli*, and one (0.2%) carbapenem-resistant *K. oxytoca* which was resistant to ERT, MEM and IMP.

### Association of fecal carriage of ESBL-E, pAmpC-E, CIP-RE and CIRE with risk factors

Statistical analysis revealed that age, gender and education did not significantly affect colonization with ESBL-E, pAmpC-E, CIP-RE or CIRE (*p* > 0.05). A statistical correlation was found between the socioeconomic status and fecal carriage of CIP-RE. Prevalence of CIP-RE carriage was higher in the participants with middle or high income (*n* = 27/192; 14.1%) compared to those with low income (*n* = 23/298; 7.7%) (*p* = 0.024, OR 1.96, 95% CI 1.09–3.53) (Table 2).

**Table 2:** Association of fecal carriage of ESBL-E, pAmpC-E, CIP-RE, and CIRE with risk factors, Northern Cyprus, 2017

Risk factors	ESBL-E			pAmpC-E			CIP-RE			CIRE		
	Positive n/N (%)	*p* value	OR (95% CI)	Positive n/N (%)	*p* value	OR (95% CI)	Positive n/N (%)	*p* value	OR (95% CI)	Positive n/N (%)	*p* value	OR (95% CI)
Age												
19–30	60/238 (25.2)	0.059	0.66 (0.43–1.02)	9/238 (3.8)	0.352	0.61 (0.21–1.74)	24/237 (10.1)	0.855	1.06 (0.60–1.89)	4/234 (1.7)	0.203	0.23 (0.03–2.10)
31 and above	46/253 (18.2)			6/256 (2.3)			27/254 (10.6)			1/249 (0.4)		
Total	106/491 (21.6)			15/494 (3.0)			51/491 (10.4)			5/483 (1.0)		
Gender												
Male	76/318 (23.9)	0.087	1.50 (0.94–2.39)	9/320 (2.8)	0.743	0.84 (0.29–2.40)	32/318 (10.1)	0.846	0.94 (0.52–1.72)	3/312 (1.0)	0.672	0.56 (0.11–2.82)
Female	31/179 (17.3)			6/180 (3.3)			19/179 (10.6)			3/177 (1.7)		
Total	107/497 (21.5)			15/500 (3.0)			51/497 (10.3)			6/489 (1.2)		
Education												
University or higher	24/116 (20.7)	0.802	0.94 (0.56–1.56)	2/118 (1.7)	0.538	0.49 (0.11–2.20)	17/116 (14.7)	0.075	1.75 (0.94–3.27)	3/114 (2.6)	0.142	3.35 (0.67–16.84)
Lower than university	83/381 (21.8)			13/382 (3.4)			34/381 (8.9)			3/375 (0.8)		
Total	107/497 (21.5)			15/500 (3.0)			51/497 (10.3)			6/489 (1.2)		
Socioeconomic status												
Middle and high	44/192 (22.9)	0.580	1.13 (0.73–1.75)	4/194 (2.1)	0.307	0.55 (0.17–1.76)	27/192 (14.1)	**0.024**	1.96 (1.09–3.53)	2/189 (1.1)	1.000	0.77 (0.14–4.26)
Low	62/298 (20.8)			11/299 (3.7)			23/298 (7.7)			4/293 (1.4)		
Total	106/490 (21.6)			15/493 (3.0)			50/490 (10.2)			6/482 (1.2)		
Presence of any GIS^a^												
Yes	17/67 (25.4)	0.411	1.28 (0.71–2.33)	4/67 (6.0)	0.128	2.44 (0.75–7.88)	8/67 (11.9)	0.626	1.22 (0.55–2.72)	3/65 (4.6)	**0.033**	6.79 (1.34–34.39)
No	90/430 (20.9)			11/433 (2.5)			43/430 (10.0)			3/424 (0.7)		
Total	107/497 (21.5)			15/500 (3.0)			51/497 (10.3)			6/489 (1.2)		
Antibiotic history^b^												
Yes	35/123 (28.5)	**0.031**	1.67 (1.04–2.67)	6/124 (4.8)	0.220	2.07 (0.72–5.95)	10/123 (8.1)	0.369	0.72 (0.35–1.48)	4/119 (3.4)	**0.033**	6.40 (1.16–35.39)
No	72/374 (19.3)			9/376 (2.4)			41/374 (11.0)			2/370 (0.5)		
Total	107/497 (21.5)			15/500 (3.0)			51/497 (10.3)			6/489 (1.2)		
Travel history^b^												
Yes	21/92 (22.8)	0.737	1.10 (0.64–1.89)	7/92 (7.6)	**0.010**	4.12 (1.45–11.66)	9/92 (9.8)	0.867	0.94 (0.44–2.00)	2/89 (2.2)	0.300	2.28 (0.41–12.62)
No	86/405 (21.2)			8/408 (2.0)			42/405 (10.4)			4/400 (1.0)		
Total	107/497 (21.5)			15/500 (3.0)			51/497 (10.3)			6/489 (1.2)		

Abbreviations: *ESBL-E* Extended-spectrum β-lactamase-producing *Enterobacteriaceae*; *pAmpC-E* Plasmid-mediated AmpC β-lactamase-producing *Enterobacteriaceae*, *CIP-RE* Ciprofloxacin-resistant *Enterobacteriaceae*, *CIRE* Carbapenem-intermediate or -resistant *Enterobacteriaceae*; *OR* Odds ratio, *CI* Confidence interval

^a^Indicates the presence of at least one gastrointestinal symptom at the time of sample collection

^b^The period covers the last six months prior to the study

Statistically significant (*p*<0.05) results were indicated in bold

In the study, presence of any GIS at the time of sample collection was statistically correlated with fecal carriage of CIRE. Prevalence of CIRE carriers having current GIS (*n* = 3/65; 4.6%) was higher than those without symptoms (*n* = 3/424; 0.7%) (*p* = 0.033; OR 6.79, 95% CI 1.34–34.39). Statistical analysis showed that, colonization with ESBL-E or CIRE was significantly affected by antibiotic use in the last six months. Rate of ESBL-E colonization in the individuals that used antibiotic (*n* = 35/123; 28.5%) was higher than those that were not exposed to antibiotics (*n* = 72/374; 19.3%) (*p* = 0.031; OR 1.67, 95% CI 1.04–2.67). Likewise, prevalence of CIRE carriage in the participants that used antibiotic (*n* = 4/119; 3.4%) was higher than nonusers (*n* = 2/370; 0.5%) (*p* = 0.033; OR 6.40, 95% CI 1.16–35.39). Furthermore, a statistical association was found between travelling abroad in the last six months and colonization with pAmpC-E. In travellers, gastrointestinal pAmpC-E colonization was more common (*n* = 7/92; 7.6%) than in nontravellers (*n* = 8/408; 2.0%) (*p* = 0.010; OR 4.12, 95% CI 1.45–11.66) (Table 2). No statistical significance was detected between travel destination and gut colonization of resistant bacteria (*p* > 0.05).

## Discussion

Colonization of the gastrointestinal tract with resistant *Enterobacteriaceae* poses a threat not only to the affected individuals, but also to the community, since antibiotic resistance genes of the gut flora can be transferred from person to person, and to the environment [16]. Therefore, it is important to determine the prevalence of intestinal colonization with resistant *Enterobacteriaceae* in the community. In the present study, 500 healthy individuals were recruited from consecutive admissions to the routine clinical laboratories of four different hospitals. Fecal carriage rates of ESBL-E, AmpC-E, CIP-RE, and CIRE were determined and the associated risk factors were analyzed.

In this study, the prevalence of ESBL-E was found to be 21.4% (Table 1). In two community-based studies in the Netherlands, this rate was reported to be 9.5% [17] and 4.7% [6]. On the other hand, a recent study from Turkey reported higher rate of ESBL-E carriage (34.3%) in the community [30]. These findings are not surprising, as the prevalence of ESBL-E carriage in Europe is lower than the other regions [15]. In the present study, rate of pAmpC-E colonization was detected to be lower (3.0%) than ESBL-E. This finding is also consistent with other reports where pAmpC carriage was observed less commonly than ESBL-E [6, 17, 30].

Prevalence of CIP-RE intestinal colonization was detected to be 10.2% in our study (Table 1). This finding is slightly higher compared to a previous study in France, where the rate of CIP-resistant *E. coli* fecal carriage was reported to be 8.0% among hospitalized patients on admission [31]. On the contrary, Steensels et al. documented higher rate (22.0%) of CIP-resistant *E. coli* carriage in the patients undergoing prostate biopsy. Use of FQs within the six months before the biopsy was found to be a risk factor, therefore this can explain the increased prevalence of colonization with FQ-resistant strains reported by Steensels et al. [18]. In the present study, due to the missing information, antibiotic names provided by the participants were excluded from the analysis, and thus, no evaluation was done on the association between previous FQ exposure and fecal carriage of CIP-resistant strains.

Rate of CIRE fecal carriage was found to be 1.2% (Table 1). Also, prevalence of CRE colonization was 0.4% (2/500) in this study. These rates are lower than those documented elsewhere. Yamamoto et al. reported that percentage of CRE carriage among hospitalized patients in Japan was 12.2% [20]. Rossini et al. documented the colonization rate of carbapenemase-producing *Enterobacteriaceae* (CPE) to be 10.2% among inpatients in Italy [32]. Unlike those studies, participants in the present report were community-dwellers and not hospitalized. Indeed, longer hospital stay was shown to be a risk factor for fecal carriage of CRE [20]. Furthermore, carbapenem resistance in *K. pneumoniae* isolates was documented at low levels previously in Northern Cyprus [24]. Taken together, all these evidence can explain the low CRE and CIRE colonization rates in the present study.

*E. coli* was the most commonly isolated species in the study. Prevalence of *E. coli* among ESBL-E, pAmpC-E, CIP-RE and CIRE species was 94.4, 86.7, 100.0, and 33.3%, respectively. This was followed by *K. pneumoniae* and other *Enterobacteriaceae* species (Table 1). Similarly in other studies, resistant *E. coli* strains were found to be responsible for the highest intestinal colonization rates, which was followed by *Klebsiella* and other enteric bacteria species [4, 20, 30, 33].

In the present study, co-existence of resistance was observed in *Enterobacteriaceae* isolates. Notably, 20.6% (*n* = 22/107) of ESBL-producing *E. coli* strains were found to be co-resistant to CIP, and a significant association was observed between CIP resistance and ESBL-production (*p* = 0.000). Previously, de Lastours et al. demonstrated that fecal carriage of ESBL-E was significantly related to colonization with CIP-resistant *E. coli* among hospitalized patients at admission [31]. Vervoort et al. reported that 63% of ESBL-E species that colonized the patients with antibiotic-associated diarrhea showed high-level FQ-resistance [14]. Co-existence of ESBL production and CIP resistance could be attributed to the transfer of resistance genes through plasmids. Studies have shown that plasmid-mediated quinolone resistance (PMQR) genes, *qnr* and *aac(6′)-lb-cr*, were detected in ESBL-producing isolates with reduced susceptibility to FQs. Moreover, the *qnr* genes were documented to be common in ESBL-E. Therefore, these findings suggest the possibility of co-transmission of ESBL and FQ resistance via plasmids [34].

In this study, two (0.4%) CIP-resistant ESBL-harbouring *E. coli* isolates were co-producers of pAmpC. This finding supports previous studies that documented fecal carriage of both ESBL- and AmpC-producing isolates [17, 35]. No carbapenem resistance was observed in any of ESBL-producing isolates. However, one ESBL-positive *E. coli* and one ESBL-positive *K. pneumoniae* isolate were intermediately resistant to ERT. Also one ESBL-harbouring *K. pneumoniae* isolate was co-producer of pAmpC and intermediately resistant to CIP and ERT. Previously, co-existence of ESBL and carbapenemase with or without AmpC production was documented by different studies [36, 37]. In our study, one *E. coli* and one *K. oxytoca* isolate with KPC and MBL-type carbapenemase, respectively, were detected, and this result is consistent with the findings reported elsewhere [38]. Reduced carbapenem susceptibility among CIRE (*n* = 6/500; 1.2%) isolates was mostly detected against ERT (resistant = 2/6, intermediate = 4/6, susceptible = 0/6), while number of the isolates that were susceptible to IMP (resistant = 1/6, intermediate = 1/6, susceptible = 4/6) and MEM (resistant = 1/6, intermediate = 0/6, susceptible = 5/6) was higher. This result is compatible with a previous report from Northern Cyprus where the resistance rate of ERT (4.6%) was higher than those of IMP (0.0%) and MEM (1.0%) in *K. pneumoniae* isolates [24].

In order to assess the possible risk factors associated with the fecal carriage of resistant *Enterobacteriaceae*, a questionnaire was applied to each participant. Statistical analysis showed that, age and gender were not significant determinants of ESBL-E, pAmpC, CIP-RE and CIRE carriage (*p* > 0.05) (Table 2). While this finding is consistent with several reports [4, 17, 20, 39, 40], it also contradicts with a few studies. Duplessis et al. found a statistical correlation between age and fecal carriage of FQ-resistant isolates. The authors stated that age can be suggestive of previous antibiotic use and hospitalization [41]. de Lastours et al. found a significant association between male sex and carriage of FQ-resistant *E. coli* in the patients treated with FQ [42]. Rossini et al. also documented a significant relation between age, male sex and CPE colonization. The authors attributed this association to the previous admission to intensive care unit (ICU) [32]. Therefore, former antibiotic use or hospital stay appear to be the major contributors to the fecal carriage of resistant bacteria.

In the study, no significant correlation was found between the level of education and fecal carriage of resistant bacteria (*p* > 0.05) (Table 2), and this result is consistent with previous reports [40]. On the contrary, Luvsansharav et al. documented a statistical association between better education status and CTX-M-type ESBL-E carriage among the individuals in the rural areas of Thailand [21]. Furthermore, we found a significantly higher rate of CIP-RE carriage among the participants with better socioeconomic status (*p* = 0.024) (Table 2). This can be explained by the fact that the indiviuals with better socioeconomic status can have easier access to health services and also over-the-counter antibiotics [21]. As a result, self-medication is associated with a higher risk of antibiotic overuse and misuse, and therefore, it increases the risk of resistance [43].

Various reports have shown that antibiotic exposure is associated with an increased risk of fecal carriage of resistant bacteria [4, 20–22]. Consistently, our study showed that antibiotic use in the last six months was a significant determinant of ESBL-E (*p* = 0.031) and CIRE (*p* = 0.033) fecal carriage (Table 2). However, pAmpC-E and CIP-RE carriage were not affected by the previous antibiotic use (*p* > 0.05), which supports previous reports [17, 39].

International travel has been shown to be a risk factor for acquiring ESBL-E and CIP-RE in rectal flora [2]. In the present study, only pAmpC-E colonization was affected by travelling abroad in the last six months (*p* = 0.010) (Table 2), which contradicts a previous report [17]. Moreover, a significant association has been demonstrated between travel to Asia, Africa and Northern America and ESBL-E carriage [4]. However, we found no correlation between travelling to Asia or Africa and fecal carriage of resistant bacteria (*p* > 0.05).

Sixty-seven (13.4%) of the study participants had at least one GIS at the time of sample collection, and the association between having a gastrointestinal complaint and CIRE colonization was found to be statistically significant (*p* = 0.033) (Table 2). Fecal carriage of resistant bacteria has also been documented in symptomatic patients by other reports. Prevalence of ESBL-E carriage was found to be 10.1% in community-dwelling patients with gastrointestinal complaints in the Netherlands, while no carbapenemase was detected [3]. In Egypt Abdallah et al. reported that 68 and five of 100 patients having community-onset gastrointestinal complaints were colonized with ESBL-E and CPE, respectively [19]. The rates of ESBL-E (25.4%) and CIRE colonization (4.6%) among the symptomatic patients in the present study are higher than in the Netherlands but lower than in Egypt. This result is not unexpected, as the antibiotic use and resistance rates in the Netherlands are low [4] in contrast to the imprudent use of antimicrobials in Egypt [19]. This suggests that, more strict regulations should be applied on antibiotic use in Northern Cyprus.

Our study has two limitations. Firstly, names of antibiotics were excluded from the analysis because of missing information, and thus the association between exposure to specific antibiotics and fecal carriage of resistant *Enterobacteriaceae* could not be evaluated. Secondly, the molecular analysis of antibiotic resistance genes could not be performed due to insufficient financial resources. In order to detect resistance by phenotypic tests, the standard two-step algorithm was performed consisting of (i) initial screening with antibiotic containing media, and (ii) further evaluation using a confirmatory test [44]. This strategy generally gives accurate results. However, phenotypic detection of ESBL can sometimes be difficult, because presence of ESBL may be masked by co-existing pAmpC β-lactamases and carbapenemases [45]. In the present study, pAmpC (*n* = 15/500; 3.0%), KPC (*n* = 1/500; 0.2%) and MBL (*n* = 1/500; 0.2%) were detected at low levels. Co-production of ESBL was detected in two pAmpC-positive isolates, while KPC- and MBL-positive isolates were found to be ESBL-negative. In these isolates, there is a possibility that presence of ESBL might have been masked by co-existing β-lactamases. Nevertheless, considering the low rates of pAmpC and carbapenemases, number of ESBL-producing isolates that were possibly misdiagnosed as negative is also very low in our study. In order to overcome these problems, phenotypic test results should also be confirmed by genotypic methods that allow molecular identification of resistant strains. Therefore, future studies are essential to elucidate the molecular basis of resistance in bacteria that colonize the gastrointestinal tract of humans in Northern Cyprus.

## Conclusion

The fecal carriage rates of ESBL-E, pAmpC-E, CIP-RE and CIRE were found to be 21.4, 3.0, 10.2 and 1.2%, respectively in this study. Notably, CIP resistance was detected in 20.6% of ESBL-producing *E. coli* isolates. Risk factors associated with the fecal carriage of resistant *Enterobacteriaceae* were identified as higher socioeconomic status (CIP-RE), presence of any gastrointestinal complaints at the time of sample collection (CIRE), antibiotic use in the last six months (ESBL-E and CIRE), and travelling abroad within six months prior to the study (pAmpC-E).

To our knowledge, this is the first study that demonstrates the fecal carriage of resistant *Enterobacteriaceae* in Northern Cyprus. The results presented here indicate that the rates of ESBL-E and CIP-RE carriage in the community are particularly alarming. The high levels of resistance suggest that control programmes should be maintained to encourage the rational use of antibiotics, and resistance should be carefully monitored in Northern Cyprus.

## Data Availability

All data generated or analysed during this study are included in this published article.

## Abbreviations

CAZ: Ceftazidime
CD: Combined disk
CI: Confidence interval
CIP: Ciprofloxacin
CIP-RE: Ciprofloxacin-resistant *Enterobacteriaceae*
CIRE: Carbapenem-intermediate or -resistant *Enterobacteriaceae*
CLA: Clavulonic acid
CLSI: Clinical and Laboratory Standards Institute
CRE: Carbapenem-resistant *Enterobacteriaceae*
CTX: Cefotaxime
DDST: Double disc synergy test
ERT: Ertapenem
ESBL: Extended-spectrum β-lactamase
ESBL-E: ESBL-producing *Enterobacteriaceae*
FOX: Cefoxitin
FQs: Fluoroquinolones
GEM: Gemifloxacin
GIS: Gastrointestinal symptom
ICU: Intensive care unit
IMP: Imipenem
KPC: *Klebsiella pneumoniae* carbapenemase
LVX: Levofloxacin
MBL: Metallo-β-lactamase
MDR-E: Multidrug-resistant *Enterobacteriaceae*
MEM: Meropenem
NOR: Norfloxacin
OFX: Ofloxacin
OR: Odds ratio
pAmpC-E: Plasmid-mediated AmpC β-lactamase-producing *Enterobacteriaceae*
